# Supplementary material for: An open-source framework for large-scale, flexible evaluation of biomedical text mining systems
Source: J Biomed Discov Collab. 2008 Jan 29;3:1. doi: 10.1186/1747-5333-3-1 (PMC2276192; doi:10.1186/1747-5333-3-1)
Supplement: Additional file 1 — Evaluation platform source code. The additional file contains the software evaluation framework discussed in this paper. The most current release can be downloaded from SourceForge.net [1]. See the accompanying README file for instructions on installation and use. Also included in the distribution is a collection of UIMA wrappers for some commonly used BioNLP tools and annotated corpora. [file 1747-5333-3-1-S1.zip › bionlp_uima_repository-1.0.1/docs/api/edu/uchsc/ccp/uima/annotation/CCPTextAnnotation_Type.html]

CCPTextAnnotation\_Type (Center for Computational Pharmacology Util/UIMA API)


|  |  |  |
| --- | --- | --- |
| **Overview** \| **Package** \| **Class** \| **Tree** \| **Deprecated** \| **Index** \| **Help** | |  |
| **PREV CLASS**   NEXT CLASS | **FRAMES**    **NO FRAMES**     **All Classes** |  |
| SUMMARY: NESTED \| FIELD \| CONSTR \| METHOD | DETAIL: FIELD \| CONSTR \| METHOD |  |


---


## edu.uchsc.ccp.uima.annotation Class CCPTextAnnotation\_Type

```
java.lang.Object
  org.apache.uima.jcas.cas.TOP_Type
      org.apache.uima.jcas.cas.AnnotationBase_Type
          org.apache.uima.jcas.tcas.Annotation_Type
              edu.uchsc.ccp.uima.annotation.CCPAnnotation_Type
                  edu.uchsc.ccp.uima.annotation.CCPTextAnnotation_Type
```

**Direct Known Subclasses:**: CCPSemanticAnnotation\_Type, CCPSyntacticAnnotation\_Type

---

``` public class CCPTextAnnotation_Type extends CCPAnnotation_Type ```

The CCP TextAnnotation extends the base annotation class to include an annotation ID, the capability for multiple annotation spans, a link to the annotator responsible for generating the annotation, membership to annotation sets, and a link to a class mention which defines the class of this annotation.
Updated by JCasGen Tue Aug 28 16:44:10 MDT 2007

---

| **Field Summary** | |
| --- | --- |
| `static boolean` | `featOkTst` |
| `static int` | `typeIndexID` |

**Fields inherited from class org.apache.uima.jcas.cas.TOP\_Type**

`casImpl, casType, casTypeCode, instanceOf_Type, jcas, ll_cas, lowLevelArrayBoundChecks, lowLevelTypeChecks, useExistingInstance`


**Constructor Summary**

`CCPTextAnnotation_Type(org.apache.uima.jcas.JCas jcas,
org.apache.uima.cas.Type casType)`
  
          initialize variables to correspond with Cas Type and Features


| **Method Summary** | |
| --- | --- |
| `int` | `getAnnotationID(int addr)` |
| `int` | `getAnnotationSets(int addr)` |
| `int` | `getAnnotationSets(int addr, int i)` |
| `int` | `getAnnotator(int addr)` |
| `int` | `getClassMention(int addr)` |
| `int` | `getDocumentSectionID(int addr)` |
| `protected  org.apache.uima.cas.impl.FSGenerator` | `getFSGenerator()` |
| `int` | `getNumberOfSpans(int addr)` |
| `int` | `getSpans(int addr)` |
| `int` | `getSpans(int addr, int i)` |
| `void` | `setAnnotationID(int addr, int v)` |
| `void` | `setAnnotationSets(int addr, int v)` |
| `void` | `setAnnotationSets(int addr, int i, int v)` |
| `void` | `setAnnotator(int addr, int v)` |
| `void` | `setClassMention(int addr, int v)` |
| `void` | `setDocumentSectionID(int addr, int v)` |
| `void` | `setNumberOfSpans(int addr, int v)` |
| `void` | `setSpans(int addr, int v)` |
| `void` | `setSpans(int addr, int i, int v)` |

**Methods inherited from class org.apache.uima.jcas.tcas.Annotation\_Type**

`getBegin, getCoveredText, getEnd, setBegin, setEnd`

**Methods inherited from class org.apache.uima.jcas.cas.AnnotationBase\_Type**

`getSofa, getView`

**Methods inherited from class org.apache.uima.jcas.cas.TOP\_Type**

`addToIndexes, checkType, getTypeIndexID, invalidTypeArg, noObjCreate, removeFromIndexes`

**Methods inherited from class java.lang.Object**

`clone, equals, finalize, getClass, hashCode, notify, notifyAll, toString, wait, wait, wait`

**Field Detail**

### typeIndexID

```
public static final int typeIndexID
```

---


### featOkTst

```
public static final boolean featOkTst
```


**Constructor Detail**

### CCPTextAnnotation\_Type

```
public CCPTextAnnotation_Type(org.apache.uima.jcas.JCas jcas,
                              org.apache.uima.cas.Type casType)
```

:   initialize variables to correspond with Cas Type and Features


**Method Detail**

### getFSGenerator

```
protected org.apache.uima.cas.impl.FSGenerator getFSGenerator()
```

:   **Overrides:**: `getFSGenerator` in class `CCPAnnotation_Type`

---


### getAnnotationID

```
public int getAnnotationID(int addr)
```

---


### setAnnotationID

```
public void setAnnotationID(int addr,
                            int v)
```

---


### getAnnotator

```
public int getAnnotator(int addr)
```

---


### setAnnotator

```
public void setAnnotator(int addr,
                         int v)
```

---


### getDocumentSectionID

```
public int getDocumentSectionID(int addr)
```

---


### setDocumentSectionID

```
public void setDocumentSectionID(int addr,
                                 int v)
```

---


### getAnnotationSets

```
public int getAnnotationSets(int addr)
```

---


### setAnnotationSets

```
public void setAnnotationSets(int addr,
                              int v)
```

---


### getAnnotationSets

```
public int getAnnotationSets(int addr,
                             int i)
```

---


### setAnnotationSets

```
public void setAnnotationSets(int addr,
                              int i,
                              int v)
```

---


### getNumberOfSpans

```
public int getNumberOfSpans(int addr)
```

---


### setNumberOfSpans

```
public void setNumberOfSpans(int addr,
                             int v)
```

---


### getSpans

```
public int getSpans(int addr)
```

---


### setSpans

```
public void setSpans(int addr,
                     int v)
```

---


### getSpans

```
public int getSpans(int addr,
                    int i)
```

---


### setSpans

```
public void setSpans(int addr,
                     int i,
                     int v)
```

---


### getClassMention

```
public int getClassMention(int addr)
```

---


### setClassMention

```
public void setClassMention(int addr,
                            int v)
```


---


|  |  |  |
| --- | --- | --- |
| **Overview** \| **Package** \| **Class** \| **Tree** \| **Deprecated** \| **Index** \| **Help** | |  |
| **PREV CLASS**   NEXT CLASS | **FRAMES**    **NO FRAMES**     **All Classes** |  |
| SUMMARY: NESTED \| FIELD \| CONSTR \| METHOD | DETAIL: FIELD \| CONSTR \| METHOD |  |


---
